# Artemin Promotes the Migration and Invasion of Cervical Cancer Cells through AKT/mTORC1 Signaling

**DOI:** 10.1155/2022/3332485

**Published:** 2022-11-26

**Authors:** Mengjing Zhu, Ling Zhou, Jian Fu, Yijin Wang, Xiaofeng Xu, Jun Wu, Xiangyi Kong, Jian Li, Zhe Zhou, Huaijun Zhou

**Affiliations:** ^1^Department of Gynecology, Nanjing Drum Tower Hospital, The Affiliated Hospital of Nanjing University Medical School, Nanjing 210008, China; ^2^Department of Gynecology, Suqian People's Hospital of Nanjing Drum Tower Hospital Group, Suqian 223800, China; ^3^Department of Gynecology, Medical School of Southeast University Nanjing Drum Tower Hospital, Nanjing 210008, China; ^4^Department of Gynecology, Lianyungang Maternal and Child Health Hospital, Lianyungang 222006, China

## Abstract

**Background:**

The neurotrophic factor Artemin (ARTN) is involved in tumor proliferation and metastasis. Nonetheless, ARTN's significance in cervical cancer (CC) has not been studied. In our study, we propose to investigate the biological function of ARTN in CC as well as its particular regulatory mechanism.

**Methods:**

Immunohistochemistry (IHC) was used to examine the degree of ARTN protein expression in CC patient tissue. Real-time PCR and Western blotting were performed to reveal related genes' levels in CC cells. The CCK-8 test, the colony formation assay, the wound-healing assay, and the transwell assay were utilized to determine the proliferation, migration, and invasion capabilities, respectively. To generate lung metastasis models, stable ARTN-expressing SiHa cells were injected into the caudal tail vein of mice. IHC was used to examine the protein levels in CC mice model tissues.

**Results:**

ARTN was overexpressed in CC tissues relative to normal cervical tissues and linked positively with lymph node metastases (*P*=0.012) and recurrence (*P*=0.015) in CC patients. In vitro, ARTN overexpression promoted the proliferation, invasion, and migration of CC cells. In contrast, the consequences of depleting endogenous ARTN were the opposite. Moreover, overexpression of ARTN increased lung metastasis of CC cells in vivo and shortened the lifespan of mice models. In addition, ARTN overexpression significantly enhanced AKT phosphorylation on Ser473 and mTOR phosphorylation on Ser2448 and promoted the epithelial-mesenchymal transition (EMT) cascade. In addition, rapamycin, a selective inhibitor of mTORC1, might rescue the EMT phenotype caused by ARTN.

**Conclusion:**

Our findings suggested that ARTN may enhance CC metastasis through the AKT/mTORC1 pathway. ARTN is anticipated to be a novel potential therapeutic target for the treatment of CC metastases.

## 1. Introduction

Cervical cancer (CC) is the fourth most prevalent cancer among women worldwide and the fourth greatest cause of cancer-related death. In 2020, it has been estimated 604,127 new cases and 341,831 fatalities worldwide [1]. Human papillomavirus (HPV) infection, familial history of cervical cancer, sexual and reproductive history, cigarette smoking, HIV infection, and socioeconomic level are the most significant risk factors for the development of CC [2]. With effective screening programs and increased use of the HPV vaccine, incidence and mortality rates of CC have been reported to have decreased over the last few decades [3]. However, the 5-year survival rate for metastatic CC is only 16.5%, whereas it is 91.5% for localized CC [4]. EMT, which stimulates tumor cell motility and invasion, has been hypothesized to initiate cancer metastasis, according to previous research [5]. The precise mechanism of EMT regulation in CC cells is still mostly unclear. Therefore, there is an unmet clinical need to comprehend the CC metastatic process.

The glial cell line-derived neurotrophic factor (GDNF) family of ligands (GFLs), which includes GDNF, neurturin, ARTN, and persephin, is a member of the transforming growth factor-*β* (TGF-*β*) superfamily and has been implicated in the progression of tumors [6, 7]. The signal of GFLs was triggered by the creation of a complex receptor system composed of four ligand-specific nonsignaling receptors, GFR*α*1-4, connected to a receptor tyrosine kinase, RET [8]. ARTN, the recently discovered member of the GFLs, consists of 113 amino acids and 7 conserved cysteine residues [9]. ARTN prefers the receptor GFR3 and activates RET to form a signaling complex for intracellular pathway control [7]. The major function of ARTN is to promote sensory neuron survival and peripheral nerve homoeostasis [10]. It also plays a distinct role in neuropathic pain and morphological alteration of the nerve [11]. Recent research examines the increasing significance of ARTN in a variety of malignancies. ARTN can enhance oncogenicity, tumor growth, and invasiveness in human malignancies, such as breast cancer, pancreatic carcinoma, endometrial carcinoma, hepatocellular carcinoma, and non-small-cell lung carcinoma [12–16]. Previous research has demonstrated that ARTN induces oncogenicity and metastasis via activation of the AKT signaling pathway [9]. ARTN can specifically control the signaling pathways involved in the EMT process [17]. Nonetheless, the role of ARTN in CC remains unclear. In the present investigation, we attempt to elucidate the biological function and molecular mechanisms underlying ARTN's role in the progression of CC.

## 2. Materials and Methods

### 2.1. Patients Samples

From 2010 to 2019, paraffin-embedded tissue samples were obtained from 88 individuals pathologically and clinically diagnosed with CC at the Nanjing Drum Tower Hospital. These tissue samples include 88 instances of CC tissue and 30 instances of normal tumor-adjacent tissue. Patient's written informed consent was obtained for research purposes, and the Medical Ethics Committee of the Nanjing Drum Tower Hospital authorized all trials involving human volunteers.

### 2.2. Immunohistochemistry (IHC) Analysis

4 mm paraffin-embedded tissue sections were baked at 60°C for 1 hour and then treated with xylene and ethanol at varying alcohol concentrations to remove the paraffin. For antigen retrieval, deparaffinized and dehydrated slides were cooked for 20 minutes at 95°C in 0.01 M citrate buffer (pH = 6.0) in a pressure cooker. 15 minutes of blocking peroxidase activity at room temperature with 3% H_2_O_2_. The sections were then treated with anti-Artemin (1 : 100 dilution, Abcam, Cambridge, United States, ab178434) at 4°C overnight. After being rinsed with phosphate-buffered saline (PBS), the slides were incubated at room temperature for 30 minutes with the secondary antibody (Typng, China) and stained with DAB reagent. Following the termination of the DAB reaction with distilled water, the slides were stained with hematoxylin and subsequently dehydrated and sealed. The expression of ARTN was examined using a microscope. Two competent pathologists reviewed and assessed the sections stained with IHC. Assign the intensity of the staining score as follows: 0 is interpreted as negative, 1 as mild, 2 as moderate, and 3 as powerful. The percentage of tumor cells that were positive was quantified as follows: 0 for no positive tumor cells, 1 for a percentage <25%, 2 for 25–50%, and 3 for ≥50%. Multiplying the intensity of staining and the proportion of positively stained cells yields the staining index score. ARTN expression was estimated for each specimen based on its staining index score, which ranged from 0 to 9. The staining patterns were defined as follows: weak (0–3), strong (4, 6, and 9). We defined the staining patterns as follows: weak (0–3) and strong (4, 6, and 9) [18].

### 2.3. RT-PCR (Real-Time PCR) Analysis

Cells from separate groups were extracted with the TRIzol reagent (Vazyme, China) according to the manufacturer's instructions. Using reverse transcription kits, total RNA was reverse-transcribed to create cDNA (Vazyme, China). Using SYBR Green Real-Time PCR Master Mix, polymerase chain reaction (PCR) in real time was performed (Vazyme, China). Primers for detecting ARTN expression have been previously published [12]. GAPDH was utilized as a source. Using 2^−ΔΔCT^, the relative expression levels were calculated.

### 2.4. Cell Culture

HeLa, SiHa, and HEK293 human cervical carcinoma cells were obtained from the American Type Culture Collection. These cells were grown in Dulbecco's modified essential medium (DMEM, Gibco) supplemented with 10% fetal bovine serum and maintained at 37°C in a humidified room with 5% CO_2_.

### 2.5. Cell Transfection and RNA Interference

To construct an overexpression vector of ARTN, PCR primers were designed as follows: F: 5′-GAGAGAGAGAATTCATGGAACTTGGACTTGGA-3′and R: 5′-GAGAGAGAGGATCCTCAGCCCAGGCAGCCGCA-3′. The open reading frame of the human ARTN precursor sequence was amplified from HeLa cells' cDNA and then subcloned into the expression vector pCDH-CMV-EF1-T2A-Puro (System Biosciences). BamHI (#R0136L, New England Biolabs) and EcoRI (#R0101V, New England Biolabs) were used to double-ligate the PCR products. A lentiviral vector expression system contains a lentiviral vector bearing the antipuromycin (antipuro) gene. Through restriction enzyme digestion and sequencing analysis, the recombinant plasmid pCDH-CMV-EF1-ARTN-puro was examined. Additionally, we create the empty pCDH-CMV-EF1-puro vector, which expresses only antipuro ability as a control. pCDH-ARTN, pCDH-control, pLP1, pLP2, and VSVG40 were cotransfected into 293T cells using Lipofectamine TM 3000 transfection reagent (Invitrogen). After 48 and 72 hours, collect the culture supernate containing virus particles from transfected 293T cells. We employ the packaged viruses to infect cervical cancer cells after preparing them to the proper density. Puromycin (Beyotime, China) was utilized to screen the stable strain in order to generate stable overexpressing cells. The company (Shanghai GenePharma) generated targeted ARTN small interfering RNA (siRNA) and siRNA-control, and the primer was previously reported [19]. All plasmids were transiently transfected into CC cells (HeLa and SiHa) using LipofectamineTM 3000 (Invitrogen) transfection reagent per the manufacturer's instructions. In a biosafety cabinet, every transfection experiment was conducted.

### 2.6. Western Blotting

In order to extract protein for Western blotting analysis, all cervical cancer cells that had been treated in various ways were lysed. Using a BCA protein assay kit (Beyotime, China), the protein concentration was determined. It was then separated using SDS-PAGE and applied to polyvinylidene fluoride membranes (Millipore, Boston, MA, USA). After being blocked in PBS with 5% fat-free milk at room temperature for an hour, the membranes were incubated with the primary antibodies at 4°C overnight. The antibodies were as follows: mouse monoclonal antibody against GAPDH (Proteintech, 1E6D9, 1 : 10,000 dilution); rabbit monoclonal antibody against Artemin (Abcam, Cambridge, MA, ab178434, 1 : 1000 dilution); rabbit monoclonal antibody against E-cadherin (Cell Signaling Technology, #3195, 1 : 1000 dilution); mouse monoclonal antibody against vimentin (Abcam, ab20346, 1 : 1000 dilution); rabbit monoclonal antibody against mTOR (Cell Signaling Technology, #2983, 1 : 1000 dilution); rabbit monoclonal antibody against phospho-mTOR (phosphor-Ser2448) (Cell Signaling Technology, #5536, 1 : 1000 dilution); rabbit monoclonal antibody against Akt (Cell Signaling Technology, #4685, 1 : 2000 dilution); and rabbit monoclonal antibody against phospho-Akt (phosphor-Ser473) (Bioworld, BS9913, 1 : 1000 dilution). The membranes were treated with secondary horseradish peroxidase after being washed four times (5 minutes each time) in PBS containing 0.1 percent Tween (HRP). The chemiluminescence imaging technology digitalized protein bands, and Image J was used to quantify them.

### 2.7. Cell Proliferation Assay

At 37°C, cells (3 × 10^3^ per well) were seeded in 96-well plates, and each group was given five parallel plates. Each well was filled with 10 *μ*l of Cell Counting Kit-8 (CCK-8, Vazyme, China) solution and incubated at 37°C for 2 hours. The absorbance at 450 nm was then measured for each sample. Using the absorbance at 24 h, 48 h, and 72 h, the cell proliferation curves were constructed.

### 2.8. Colony Formation Assay

200 cells were seeded in six-well plates with DMEM supplemented with 10% FBS and grown at 37°C with 5% CO_2_. 14 days later, we witnessed the creation of macroscopic colonies. The surviving cells were fixed in methanol for 15 minutes and stained with 0.1% crystal violet after being rinsed with PBS. Using an inverted phase-contrast microscope, the colonies were photographed and tallied.

### 2.9. Wound-Healing Migration and Matrigel Invasion Assays

For wound-healing migration tests, HeLa and SiHa transfected cells were planted in six-well plates (1 × 10^6^ cells/well). The cell monolayer was mechanically disrupted using a sterile 200 *μ*l pipette tip to create a linear wound after serum starvation for one night. The monolayer had reached 70%–80% confluence. We observed the rate of wound closure at 0 hours, 24 hours, and 48 hours in DMEM without serum. Using Image J, the average distance traveled by the cells was determined. 2,000 cells were resuspended in DMEM without serum and sown in the upper chamber of 8 *μ*M transwell filters (Merck Millipore) for Matrigel invasion experiments. The invasion assay was performed using filters precoated in eleven-fold diluted Matrigel (BD Bioscience, San Jose, CA). The lower compartment was supplied with DMEM supplemented with 10% FBS, and the cells were incubated for 24 hours. 1 × 10^5^ cells in serum-free DMEM were seeded on a polycarbonate filter membrane devoid of 8 *μ*M polyvinylpyrrolidone. Invading cells were quantified after crystal violet staining.

### 2.10. Xenograft Mouse Metastatic Model

The development of a mouse model of CC has been described [5]. Twelve 5-week-old female BALB/c A-node mice were obtained from the Laboratory Animal Center of the Drum Tower Hospital affiliated with Nanjing University School of Medicine (Nanjing, China). Randomly dividing these mice into two groups, SiHa cells (2 × 10^6^ cells/100 *μ*l PBS) containing either ARTN or ARTN-control were injected via the caudal tail vein. After eight weeks, all mice were euthanized in preparation for the next test. The lungs of mice were sectioned serially and independently inspected by 2 expert pathologists.

### 2.11. Bioinformatic Analysis

The TCGA-CESC project was analyzed using UALCAN (https://ualcan.path.uab.edu) based on TCGA level 3 RNA-seq expression [20]. Meanwhile, according to the GTEx database, ARTN expression levels were analyzed in uterine corpus CC tissues and healthy cervical tissues (https://www.xiantao.love).

### 2.12. Statistical Analysis

SPSS version 24.0 was used to conduct statistical analysis (SPSS, Chicago, IL, USA). The connection between ARTN expression and the clinicopathological characteristics of CC patients was evaluated using the *χ*^2^ test. The Student's *t*-test or one-way ANOVA was used to assess comparisons between two groups. Using the Kaplan–Meier method and log-rank test, a survival analysis was plotted. *P* < 0.05 was deemed to be significant. All tests were conducted separately 3 times.

## 3. Results

### 3.1. ARTN Is Highly Expressed in Cervical Cancer Tissues

**Figure 1:**
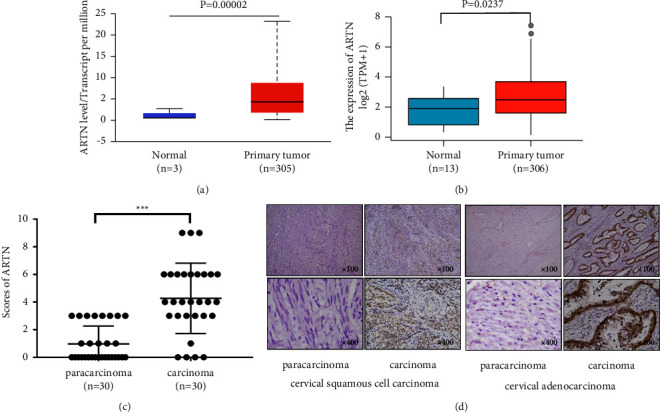
The expression of ARTN protein is increased in CC tissues. (a) According to the TCGA-CESC project and the GTEx database, ARTN expression levels were significantly greater in uterine corpus CC tissues than in healthy cervical tissues (https://ualcan.path.uab.edu, *P*=0.00002, https://www.xiantao.love, *P*=0.0237). (b) IHC analysis showing that the staining of the ARTN protein was significantly greater in paraffin-embedded specimens of CC sample tissues (*n* = 30) than in paracancerous tissues (*n* = 30, *P*=0.0005). (c) IHC staining for paracancerous tissues and squamous cell carcinoma (as seen by keratin pearls). IHC staining for paracancerous tissues and adenocarcinoma (shown by clearly distinct glands) (originally 400 and 100 magnified).

TCGA-CESC expression data and the GTEx database suggest that ARTN expression was higher in CC tissues than in normal cervical tissues (Figure 1(a), *P*=0.00002, *P*=0.0237). ARTN levels were higher in cervical squamous cell carcinoma and cervical adenocarcinoma tissues than in paracarcinoma tissues, according to IHC results (Figures 1(b)-1(c)). To further comprehend the clinical foundation driving the tissue-specific differential response to ARTN overexpression, we investigated the correlation between ARTN expression and the clinicopathological characteristics of CC patients. ARTN expression was strongly related to lymph node metastases (LNM) (*P* = 0.012) and recurrence (*P* = 0.015) in cervical cancer (Table 1).

### 3.2. ARTN Promotes Proliferation of CC Cells

**Figure 2:**
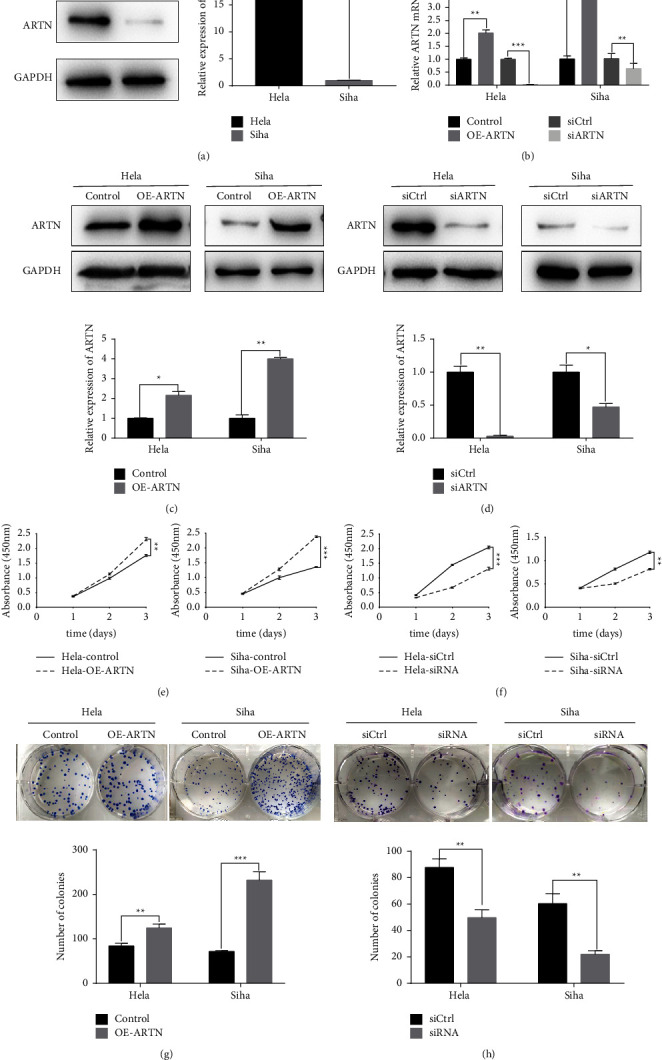
ARTN promotes proliferation of CC cells in vitro. (a) Western blot analysis showing that HeLa and SiHa cells have ARTN protein (^*∗∗∗*^*P* < 0.001). (b) In HeLa and SiHa cells that had been transfected with the lentivirus, real-time PCR demonstrated the relative ARTN mRNA expression levels (^*∗∗*^*P* < 0.01, ^*∗∗∗*^*P* < 0.001). (c)-(d) Western blot analysis revealed the ARTN protein level in HeLa and SiHa cells following transfection with produced lentivirus (^*∗*^*P* < 0.05, ^*∗∗*^*P* < 0.01). (e)-(f) The CCK-8 assays performed to detect the effects of ARTN overexpression and knockdown on the proliferation of HeLa and SiHa cells (^*∗∗*^*P* < 0.01, ^*∗∗∗*^*P* < 0.001). (g)-(h) Colony formation assays conducted following ARTN overexpression and knockdown in HeLa and SiHa cells (^*∗∗*^*P* < 0.01, ^*∗∗∗*^*P* < 0.001). The colon counts from three independent experiments are represented by error bars.

HeLa cells had more endogenous ARTN protein expression than SiHa cells (Figure 2(a)). Both HeLa and SiHa cells were engineered to express ARTN in a stable manner, and endogenous ARTN was silenced using siRNA. RT-PCR revealed that the ARTN mRNA level in HeLa and SiHa cells was dramatically boosted or decreased (Figure 2(b)). We discovered that ARTN protein expression rose or decreased considerably in the two cell lines, respectively (Figures 2(c)-2(d)). The CCK-8 assay demonstrated that ARTN overexpression significantly increased cellular proliferation (Figure 2(e)). In contrast, blocking ARTN expression with siRNA significantly reduced cell proliferation (Figure 2(f)). Similarly, colony formation experiments confirmed that overexpression of ARTN increased cell colony-forming ability, but the silencing of ARTN dramatically decreased cell colony-forming ability (Figures 2(g)–2(h)). These results revealed that ARTN improved the proliferative capacity of CC cells.

### 3.3. ARTN Promotes Migration and Invasion of CC Cells

**Figure 3:**
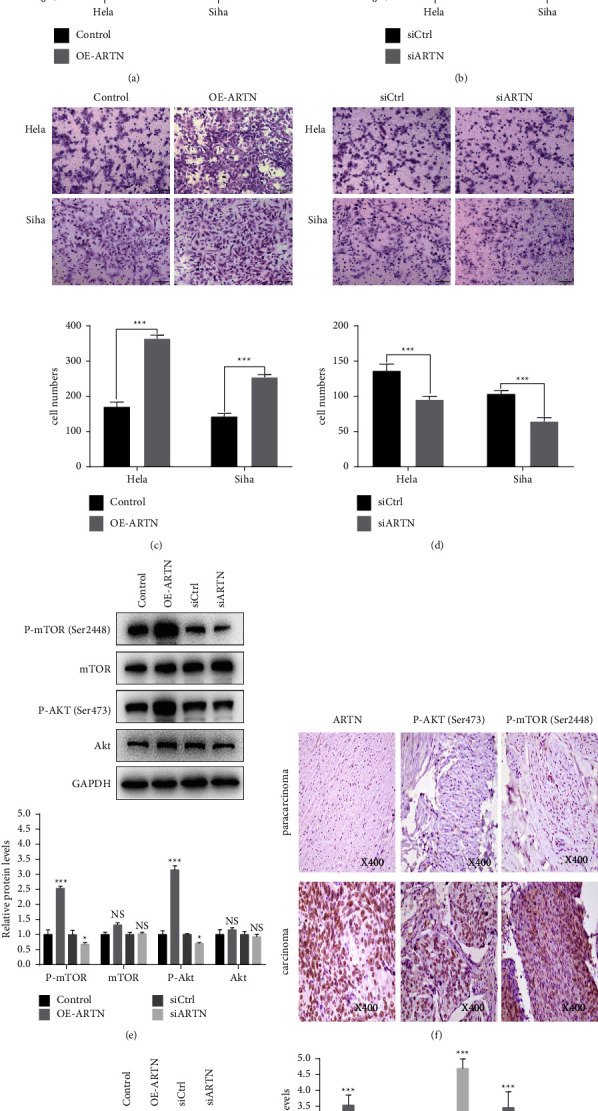
AKT/mTOR-mediated EMT is activated by ARTN, which then encourages CC cell migration and invasion. (a and b) The effects of the treatments on cell migration detected using wound-healing migration assays. The colon counts from three separate studies are represented by error bars (^*∗∗∗*^*P* < 0.001). (c and d) Transwell assays used to determine the invasion abilities. The colon counts from three separate studies are represented by error bars (scale bar, 200 *μ*M, ^*∗∗∗*^*P* < 0.001). (e) IHC staining showing that CC tissues with ARTN and paracancerous tissues had relative P-AKT and P-mTOR protein levels (original magnification, ×400). (f) After ARTN was overexpressed and knocked down in HeLa and SiHa cells, a Western blot revealed the relative amounts of AKT and mTOR protein (^*∗∗*^*P* < 0.01, ^*∗∗∗*^*P* < 0.001). (g) Western blot revealing the relative EMT protein levels after overexpression and knockdown of ARTN in HeLa and SiHa cells (^*∗*^*P* < 0.05, ^*∗∗*^*P* < 0.01, ^*∗∗∗*^*P* < 0.001).

The effect of ARTN on the migration of CC cells was investigated using wound-healing tests. Our results demonstrated that overexpression of ARTN enhanced the motility of HeLa and SiHa cells. Overexpression of ARTN in HeLa and SiHa cells significantly accelerated the healing of scratch wounds compared to control cells (Figure 3(a)). Similarly, siRNA-ARTN inhibited the filling of the migratory region in HeLa and SiHa cells (Figure 3(b)). We then conducted transwell experiments to test the invasiveness of CC cells. Both HeLa and SiHa cells exhibited a greater number of invasive cells with ARTN overexpression than the control cells (Figure 3(c)). ARTN knockdown decreased the amounts of invading cells in two cell lines (Figure 3(d)). In vitro, our findings indicated that ARTN increases the migration and invasion of CC cells.

### 3.4. mTOR Activation by ARTN Is Mediated by AKT/mTORC1

Through the regulation of many cellular and molecular functions, the AKT/mTOR signaling pathway is essential for tumor initiation, invasion, and metastasis and plays a key role in the development of CC [21, 22]. We question whether ARTN promotes cancerous tendencies in CC cells via this signaling pathway. As shown in Figures 3(e)-3(f), ARTN activated the AKT/mTOR signaling pathway by raising the phosphorylation of AKT and mTOR in vivo and in vitro. mTOR is a member of the PI3K-related kinase family and consists of two different protein complexes, mTOR Complex 1 (mTORC1) and 2 (mTORC2), with mTORC1 being susceptible to rapamycin treatment. Traditionally, the PI3K/AKT pathway is upstream of mTORC1 [23]. mTOR was phosphorylated at Ser2448 via the PI3K/AKT signaling pathway, and Ser2448 phosphorylation is regarded as a rapamycin-sensitive marker [24]. Consequently, we came to the conclusion that ARTN could increase the advancement of CC via AKT/mTORC1 signaling.

### 3.5. Rapamycin Reverses ARTN-Induced EMT of HeLa Cells

**Figure 4:**
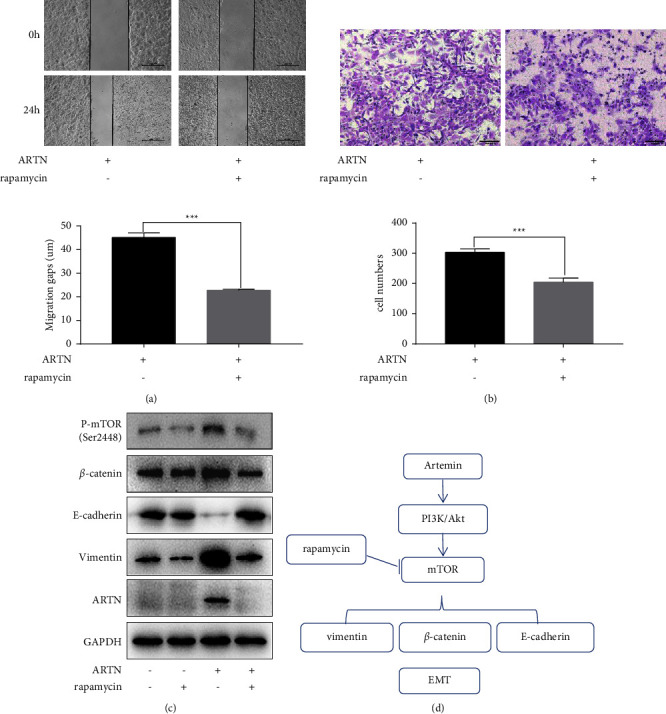
Rapamycin reverses the EMT that ARTN causes in CC cells. (a) The effects of the treatments on SiHa cells' migration using wound-healing migration assays. “ARTN+” means that ARTN was overexpressed in CC cells, and “rapamycin+” means that rapamycin was added to the culture medium of CC cells. The colon counts from three separate studies are represented by error bars (^*∗∗∗*^*P* < 0.001). (b) Transwell invasion experiments utilized to assess the ability of SiHa cells to invade the following treatment. The colon counts from three separate studies are represented by error bars (scale bar, 200 *μ*M, ^*∗∗∗*^*P* < 0.001). (c) The relative P-AKT, P-mTOR, and EMT protein levels displayed on a Western blot. (d) A simplified model showed that ARTN encourages CC cell migration and invasion.

As EMT has garnered substantial attention as a conceptual framework for explaining metastatic and invasive behavior during the course of cancer [25], we identified EMT-related hallmark proteins in CC cells. ARTN raised the expression of vimentin and *β*-catenin while decreasing the expression of E-cadherin, as depicted in Figure 3(g). We hypothesized that ARTN-induced EMT contributes to CC cell invasion and migration. According to a previous study, stimulation of mTOR signaling induces EMT to enhance the invading capacity of carcinoma cells [26]. Consequently, we examined whether the mTOR pathway is involved in the ARTN-induced EMT process. Rapamycin is a selective inhibitor of mTORC1, and we investigated its effect on SiHa cells stimulated by ARTN. As demonstrated in Figure 4(a), rapamycin administration reduced the migration gaps, and rapamycin may reverse the impact of ARTN. In addition, transwell experiments demonstrated that the number of invasive cells with ARTN overexpression was considerably lower in the presence of rapamycin than in its absence (Figure 4(b)). ARTN increased the expressions of P-Akt (Ser473), P-mTOR (Ser2448), vimentin, and catenin while decreasing the expression of E-cadherin, as revealed by Western blot (Figure 4(c)). Moreover, Western blot analysis indicated that rapamycin therapy decreased P-AKT (Ser473) and P-mTOR (Ser2448) expression levels, which were involved in the ARTN-induced EMT progression (Figure 4(c)). In response to ARTN-driven EMT, which is triggered via the AKT/mTORC pathway, rapamycin suppresses the migration and invasion of CC cells (Figure 4(d)).

### 3.6. ARTN Promotes CC Metastasis In Vivo

**Figure 5:**
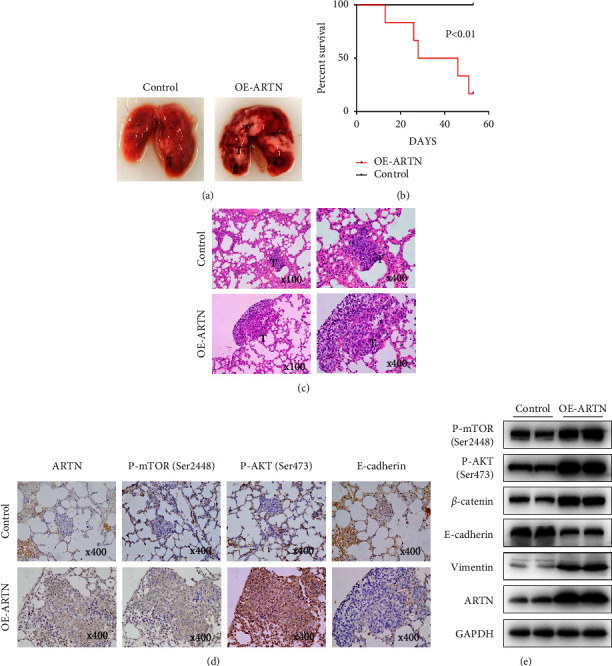
Through AKT/mTORC1 signaling, ARTN accelerated the metastasis of CC cells in vivo. (a) “T” denotes metastatic nodules on the lungs images, which were taken from mouse models of metastasis. (b) Kaplan–Meier survival analysis of 12 mice injected with oe-ARTN or ARTN-control SiHa cells by the lateral tail vein (*P* < 0.01, log-rank test). (c) The lung tissues of mice stained with H&E (original magnifications, ×100 and ×400). (d) In lung sections, IHC staining was performed for ARTN, P-mTOR, P-AKT, and E-cadherin (original magnifications, ×400). (e) Western blot analysis of lung tissues revealing the presence of P-mTOR, P-AKT, vimentin, *β*-catenin, E-cadherin, and ARTN protein expressions.

To further investigate whether ARTN enhances CC metastasis in vivo, we developed lung metastasis models by injecting stably-expressing SiHa cells into mice via the caudal tail vein. The CC mouse model was developed as described in [5]. Eight weeks after implantation, every mouse was euthanized. Further examination of these mice's vital organs revealed that the number of lung metastatic nodules in the ARTN overexpression group was much higher than in the control group (Figure 5(a)). We noticed that mice in the ARTN overexpression group had a shorter lifespan (Figure 5(b)). As previously observed, the liver and spleen also exhibited severe abnormalities [15]. In addition, H & E staining indicated that the nodules in the lungs of mice were metastatic tumors, and an IHC assay was conducted on these nodules in two groups of mice. In comparison to control tumors, nodules with overexpressed ARTN had higher expression of P-AKT and P-mTOR and decreased expression of E-cadherin, as determined by IHC (Figures 5(c)-5(d)). Western blot analysis also revealed that ARTN overexpression enhanced the expression of P-AKT, P-mTOR, vimentin, and *β*-catenin while decreasing the expression of E-cadherin (Figure 5(e)). These data suggested that ARTN overexpression increased the metastatic potential of CC cells in vivo via AKT/mTORC1 signaling.

## 4. Discussion

Cervical cancer is the most prevalent gynecologic malignancy, and cervical cancer metastasis is one of the primary causes of death and refractory disease. ARTN is a protein that is highly conserved among mammalian species. Molecularly, the three-dimensional structure of ARTN resembles that of GDNF and NRTN, and it shares some similarity with members of the TGF-*β* family. ARTN mRNA expression was controlled at the transcriptional level by the master activator protein 1 (AP-1) transcription factor, c-Jun [8]. Increased ARTN expression by tumor cells is an essential marker of cancer progression, as autocrine and paracrine processes may enhance invasiveness, metastasis, carcinogenesis, and drug resistance to certain chemotherapies [9]. Previous research indicated that ARTN enhances metastasis and invasiveness in breast cancer, pancreatic cancer, endometrial cancer, and non-small cell lung cancer. Through expression of twist family BHLH transcription factor 1 (TWIST1), ARTN increases metastasis in patients with estrogen receptor (ER)-negative mammary cancer (ER-MC) [17]. As a master regulator of morphogenesis and a TGF- cooperation factor, Twist1 plays a crucial role in tumor metastasis [27]. ARTN, a member of the TGF-*β* superfamily, may control TWIST1 to promote EMT and metastasis, hence promoting cell motility and invasion [17]. In addition, ARTN regulates the cancer stem cell population (CSC-like cell population), which has the ability to promote tumor-initiating capacity and increase radio- and chemo-resistance, which may be responsible for metastasis and palindromia [13]. Results from a study of pancreatic adenocarcinoma indicate that ARTN may have a significant role in tumor metastasis, particularly perineural invasion (PNI). The possible mechanism for this phenomenon is that ARTN increases CXC chemokine receptor 4 (CXCR4) expression via AKT and ERK1/2/NF-KB signaling and the SDF-1*α*/CXCR4 axis [19]. Higher ARTN expression in endometrial carcinoma (EC) was associated with enhanced invasiveness, a higher tumor grade, and lymphatic metastasis. Consistently, ARTN stimulates resistance to doxorubicin and paclitaxel under the particular control of CD24 poses a challenging therapeutic obstacle in the treatment of recurrent and metastatic EC [28]. ARTN was discovered to promote the migration and invasion of non-small-cell lung cancer (NSCLC) cells by upregulating BCL2 expression [16]. In our investigation, the protein level of ARTN was shown to be elevated in CC tissues compared to those adjacent to normal tumors. The correlations between ARTN expression and clinicopathological characteristics, as well as the degrees of ARTN's effect on the cellular biological function of CC cells, were investigated further. First, by combining IHC results with clinical data analysis, we determined that higher ARTN expression was substantially linked to LNM and recurrence. We then explored the functions of ARTN in CC cells by overexpressing or inhibiting ARTN. Increasing ARTN stimulated CC cell proliferation, invasion, and migration. These data support the oncogene role of ARTN in cancer, as shown by prior studies [12, 29]. Lastly, we investigated whether ARTN overexpression improved the metastatic potential of CC cells in vivo.

Activated mTOR signaling (activating somatic mutations of mTOR) plays a vital function in response to cell survival, invasion, metastasis, antiapoptosis, and inhibition of autophagy [30]. mTOR is composed of two different signaling complexes, mTORC1 and mTORC2. The components of mTORC1 are mTOR, Raptor, mLST8, and PRAS40. When the FKBP12-rapamycin combination binds to the FRB domain, the recruited substrate by the Raptor entrance might be inhibited, resulting in mTORC1 kinase inhibition [31]. The mTOR2 is composed of mTOR, MLST8, mSIN1, Protor, and Rictor (mTOR's rapamycin-resistant companion) [32]. It has been demonstrated that the growth factor-mediated RTKs (receptor tyrosine kinases)/PI3K/Akt signaling pathway is a key upstream signaling pathway of the mTOR protein molecule [33]. It has been observed that stimulation of mTOR signaling produces EMT to enhance renal cancer cell invasion [34]. Lamellipodia are formed when cancer cells undergo a considerable remodeling of the actin cytoskeleton during the EMT process [35]. The TGF-*β* signaling pathway may activate the mTOR signaling pathway, resulting in EMT induction [36]. The TGF-*β* signaling pathway phosphorylates AKT directly at Ser473 and controls the downstream effector mTORC2 to produce EMT [37]. Unknown is, however, the role of mTORC1 in this process. As a member of the TGF-*β* family, we observed that ARTN regulates the PI3K/AKT/mTORC1 pathway and influences the migration and invasion of CC cells. Concomitantly with our findings, several observations in this study supported our views. First, ARTN-overexpressing CC cells had substantially active Akt (phospho-Ser473) and mTOR phosphorylation at serine residues 2448 compared to the control group. Second, ARTN expression is connected with the dysregulation of EMT-related proteins, including vimentin, *β*-catenin, and E-cadherin. In the course of cancer, these protein levels indicate that carcinoma cells acquire mesenchymal traits and lose epithelial ones [38]. Lastly, rapamycin, an inhibitor of mTORC1, can suppress ARTN-induced AKT and mTOR phosphorylation and reverse the expression of EMT-related proteins. Therefore, we concluded that ARTN induces an increase in migration and invasion through regulation of AKT/mTORC1 signaling. Consistent with our findings, ARTN stimulated AKT signaling and accelerated the migration and invasion of mammary carcinoma and pancreatic adenocarcinoma cancer cells [17]. Han et al. demonstrated that ARTN stimulates PI3K pathways and promotes the advancement of hepatocellular carcinoma by promoting caspase-9 Thr125 phosphorylation, which is essential for inhibiting caspase-9 activation and apoptosis initiation [15]. Whether or not mTOR mediates this development has yet to be determined.

In conclusion, the level of ARTN is higher in CC tissues and cells, and suppression of ARTN reduced the progression of CC by inhibiting the AKT/mTORC1 pathway and targeting EMT. Recently, it was discovered that elevated serum ARTN in hepatocellular carcinoma patients is associated with a poor prognosis [15]. As is common knowledge, squamous cell carcinoma-related antigens (SCC) are released by squamous cancer cells and are valuable for monitoring recurrence and assessing the efficacy of chemotherapy for CC [39]. Nonetheless, the sensitivity of SCC in cervical adenocarcinoma and other kinds of cervical cancer is inadequate [40]. The serum ARTN in CC patients and the link between serum ARTN and CC deserve additional research.

## Figures and Tables

**Table 1:** The correlation between ARTN expression and clinicopathologic characteristics in IHC analysis^a^.

Variables	Number (*n* = 88)	ARTN expression		*P* value
		Week (%)	Strong (%)	
Age (years)				0.747
<55	64	21 (32.8)	43 (67.2)	
≥55	24	7 (29.2)	17 (70.8)	
Histological type				0.834
Squamous carcinoma	61	19 (31.1)	42 (68.9)	
Adenocarcinoma	19	6 (31.6)	13 (68.4)	
Others	8	3 (37.5)	5 (62.5)	
FIGO stage				0.988
I	75	24 (32.0)	51 (68.0)	
II	13	4 (30.8)	9 (69.2)	
Tumor size (cm)				0.866
≤4	67	21 (31.3)	46 (68.7)	
>4	21	7 (33.3)	14 (66.7)	
Pathological grade				0.122
G1	13	7 (53.8)	6 (46.2)	
G2	48	14 (29.2)	34 (70.8)	
G3	27	7 (25.9)	20 (74.1)	
Lymph node metastases				0.012^*∗*^
Yes	21	2 (8.3)	19 (90.5)	
No	67	26 (38.8)	41 (61.2)	
Recurrence				0.015^*∗*^
Yes	16	1 (6.3)	15 (93.8)	
No	72	27 (37.5)	45 (62.5)	

FIGO, The International Federation of Gynecology and Obstetrics. ^a^Values are given as a number (percentage), unless indicated otherwise.

## Data Availability

The [DATA TYPE] data used to support the findings of this study are included within the article.
